# Isoform Sequencing Provides Insight Into Freezing Response of Common Wheat (*Triticum aestivum* L.)

**DOI:** 10.3389/fgene.2020.00462

**Published:** 2020-06-11

**Authors:** Xingwei Zheng, Mengmeng Shi, Jian Wang, Na Yang, Ke Wang, Jilong Xi, Caixia Wu, Tianyuan Xi, Jun Zheng, Jiancheng Zhang

**Affiliations:** ^1^Institute of Cotton Research, Shanxi Agricultural University, Yuncheng, China; ^2^Institute of Wheat Research, Shanxi Agricultural University, Linfen, China

**Keywords:** wheat (*Triticum aestivum* L.), freezing, transcriptome, full-length transcript, alternative splicing

## Abstract

The objective of the study is to reveal the freezing tolerance mechanisms of wheat by combining the emerging single-molecule real-time (SMRT) sequencing technology PacBio Sequel and Illumina sequencing. Commercial semiwinter wheat Zhoumai 18 was exposed to −6°C for 4 h at the four-leave stage. Leaves of the control group and freezing-treated group were used to perform cDNA library construction. PacBio SMRT sequencing yielded 51,570 high-quality isoforms from leaves of control sample of Zhoumai 18, encoded by 20,366 gene loci. In total, 73,695 transcript isoforms, corresponding to 23,039 genes, were identified from the freezing-treated leaves. Compared with transcripts from the International Wheat Genome Sequencing Consortium RefSeq v1.1, 57,667 novel isoforms were discovered, which were annotated 21,672 known gene loci, as well as 3,399 novel gene loci. Transcriptome characterization including alterative spliced events, alternative polydenylation sites, transcription factors, and fusion transcripts were also analyzed. Freezing-responsive genes and signals were uncovered and proved that the ICE-ERF-COR pathway and ABA signal transduction play a vital role in the freezing response of wheat. In this study, PacBio sequencing and Illumina sequencing were applied to investigate the freezing tolerance in common wheat, and the transcriptome results provide insights into the molecular regulation mechanisms under freezing treatment.

## Introduction

Wheat (*Triticum aestivum* L.) is globally the third-largest food crop, next to maize and rice. It provides approximately 20% of human energy and is richer in protein and fat than other crops. Millions of tons of grain yield are lost every year due to cold (Winfield et al., 2010). Winter wheat can acquire cold tolerance through cold acclimation and vernalization, which usually takes ∼4 to 8 weeks, and during this period, plants can adapt to the gradually changing temperature. However, in recent years, warm winter and chilling in late spring occurred frequently; plants in key vegetative stages can be exposed to a very sudden subzero temperature when the winter-hardiness is almost lost (Li et al., 2015).

The molecular basis of plant cold tolerance has been widely analyzed. Previous studies have shown that the cold acclimation is an adaptation process involving multiple biochemical and physiological changes, which regulated by the induced expression or repression of a series of regulatory and functional genes (Li et al., 2015; Ma et al., 2015; Mao et al., 2019). Understanding the molecular mechanisms in the cold response is important to improve cold tolerance using molecular techniques (Rubio et al., 2018; Ye et al., 2019). Transcriptome sequencing has been proven to be an efficient method for cold-related gene discovery, which has been studied extensively in *Arabidopsis* (Lee et al., 2005) and cereal crops such as rice (Zhang et al., 2012), maize (Trzcinska-Danielewicz et al., 2009), barley (Tommasini et al., 2008), and wheat (Gulick et al., 2005). RNA-seq has discovered several cold-related pathways and a series of key cold-responsive genes (Laudencia-Chingcuanco et al., 2011; Li et al., 2018; Pu et al., 2019).

Bread wheat is an allohexaploid species with a complex genome of approximately 17 giga-base pairs (Gbp) and has a repeat content of approximately 80%. Because of the limitation of sequencing read length, the transcript structure obtained by splicing is not complete, and the third-generation sequencing technology represented by PacBio effectively solves this problem. The platform utilizes single-molecule real-time (SMRT) sequencing technology, also known as SMRT sequencing (Xiao et al., 2018b). With its long length read advantage, it can directly obtain high-quality full-length transcript information without interruption or assembly. The reference genome sequence of hexaploid bread wheat cv. Chinese spring (CS) with 21 chromosomal assembly and gene annotation [International Wheat Genome Sequencing Consortium (IWGSC) RefSeq v1.1] has been presented by the IWGSC (Appels et al., 2018). Wheat researchers aimed to complement the reference genome and transcriptome information using the PacBio SMRT technology and made significant findings involved in grain development (Dong et al., 2015) and heat sensing and signaling (Wang et al., 2019). In order to uncover the freezing adaptation process in wheat, we sequenced the transcripts expressed during freezing stress in common wheat using the SMRT sequencing platform PacBio Sequel and Illumina short reads sequencing, in order to assess more valuable data to complement current common wheat genome annotation and cold-resistant transcriptome research.

## Materials and Methods

### Plant Materials and Treatments

The common wheat (*T. aestivum* L.) cultivar Zhoumai 18 was used in the current study. The cultivar was a widely adapted semiwinter wheat genotype grown in Southern Huang-Huai wheat area. Twenty pots (inner diameter 20 mm, height 25 mm) filled with the same amount of soil were buried in experimental field in Linfen, Shanxi (36°06′24″ N, 111°30′55″ E). Ten seeds were planted in each pot at conventional summer-fallow period on October 2, 2018. Thirty days later, pots were gotten back, and seedlings of Zhoumai 18 were treated at −6°C for 0, 4, 6, and 8 h with five replicates. Fully expanded leaves with the same leaf age were collected from eight plants for each replicate and stored at −80°C for RNA extraction.

### Leaf Cell Plasma Membrane Permeability Assay

The relative electrolyte leakage was measured with fresh materials immediately after freezing treatment finished. Clean leaves were stored in wet gauze to prevent the leaves from losing water and were cut into 1-cm pieces. Each treatment was divided into two groups, each group was repeated three times with 0.5 g sample, which was immersed with 20 mL of ddH_2_O. A set of samples (S1) was placed in a vacuum desiccator, and the gas was repeatedly pumped three to four times with a vacuum pump to remove air between the water and the surface of the blade and between the cell gaps, so that the electrolyte in the leaf tissue was easily oozing out. The pressure was controlled at 400 to 500 mm Hg to make the decompression conditions uniform. After 0.5 h of decompression, the normal pressure was restored, and the temperature was kept at 20°C to 30°C for 2 to 3 h. Another set of samples (S2) was placed in a boiling water bath for 10 to 15 min to kill the tissue and completely destroy the plasma membrane. The tissue exudates of the two groups of samples were separately poured into clean small glasses, and the conductivity was measured by a conductivity meter Starter 3100C (OHAUS, Newark, NJ, United States).

$$\text{Relative}\,\text{electrolyte}\,\mathrm{leakage}=\frac{\text{electrolyte}\,\text{leakage}\,\text{of}\,\mathrm{S1}}{\text{electrolyte}\,\text{leakage}\,\text{of}\,\mathrm{S2}}\times 100\%$$

### RNA Sample Preparation, Library Preparation, and Sequencing

Total RNA was isolated using RNAprep Pure Plant Kit (Tiangen Biotech, Beijing, China) and treated with RNase-free DNase I (Takara, Dalian, China) to remove genomic DNA contamination. RNA integrity was evaluated with a 1.0% agarose gel stained with ethidium bromide. Thereafter, the RNA purity and concentration were assessed using a SpectraMax^®^ QuickDrop^TM^ Micro-Volume Spectrophotometer (Molecular Devices, San Jose, CA, United States) and an Agilent 2100 Bioanalyzer (Agilent Technologies, Santa Clara, CA, United States). Samples with the RNA integrity number greater than 8.0 were used for cDNA library construction and sequencing. RNA of three biological replicates after 4-h freezing treatment were sequenced using Illumina MiSeq platform with paired-end (2 × 150 bp) sequencing, and the RNAs from each replicate were mixed in equal volume and sequenced using the PacBio Bioscience Sequel platform.

Illumina library construction: mRNA with poly-A was isolated from the total RNA using oligo(dT)-attached magnetic beads and then broken into fragments of approximately 300 bp. After cDNA synthesis, sequencing libraries with 300- to 400-bp fragments were generated by polymerase chain reaction (PCR) amplification, and then the library quality was tested with Agilent 2100 Bioanalyzer.

PacBio library preparation and SMRT sequencing: total RNA was reversely transcribed into cDNA using Clontech SMARTer reaction, and then large-scale PCR was conducted to access sufficient total cDNA. After exonuclease digestion and removal of unligated sequences at both ends of cDNA, a complete SMRT bell library was formed. The library was qualified and sequenced using the PacBio Bioscience Sequel platform based on the effective concentration of the library and the data output requirements.

### PacBio Data Quality Filtering and Isoform Detection

The original Iso-seq data were preprocessed using the PacBio SMRT-link v7.0 software requiring parameters min-length = 50, max-length = 15,000, and min-readScore = 0.75. The circular consensus sequences (CCSs) were generated from the correction of subreads with the parameters min-SubreadLength = 50, min-Passes = 1, and min-PredictedAccuracy = 0 (Xiao et al., 2018a). According to whether the sequence contains 5′ primers, 3′ primer and the poly(A) tail, the CCSs were divided into full-length and non–full-length sequences. Only the CCS with all three signals and without any adapter sequences was considered as a full-length non-chimeric (FLNC) read. Then, the consensus sequences were obtained by clustering the full-length reads using the algorithm hierarchical n^∗^log(n). Further polishing with the consensus sequences was performed using Quiver to obtain high-quality consensus reads for subsequent analysis.

Full-length consensus reads were first mapped to the IWGSC RefSeq v1.1 using the Genome Mapping and Alignment Program (GMAP, version: 2017-06-20, Wu et al., 2005) with the following parameters: -no-chimeras, –expand-offsets 1, -B 5, -f samse, −n 1. Next, indels and mismatches were corrected using the reference genome. Finally, transcripts unmapped to the reference sequence were conducted BLAST search (*E* value ≤ 10^−5^) against NCBI non-redundant, NCBI nucleotide sequence (NT), Pfam, Cluster of Orthologous Groups (KOG/COG), SwissProt, Kyoto Encyclopedia of Genes and Genomes (KEGG), and Gene Ontology (GO) databases.

### Analysis of the Genes and Transcripts in Response to Freezing Shock

Adapters and poor-quality data in Illumina short reads were filtered, and the clean data were mapped to the wheat reference genome (IWGSC RefSeq v1.1) using TopHat2 (Kim et al., 2013). HTSeq software (Anders et al., 2015) was used to calculate the expression values for each gene in terms of FPKM (fragments per kilo bases per million fragments). The differentially expressed genes (DEGs) between the control and freezing samples were identified using DESeq package (Anders and Huber, 2010). Differentially expressed genes are determined by |log_2_ fold change|≥1 and false discovery rate (FDR)-adjusted *p* ≤ 0.05.

TopGO was used for GO analysis to determine the main biological functions performed by DEGs. The KEGG pathway mapping was used to indicate the location of the DEGs. The hypergeometric distribution test with *p* ≤ 0.05 determined statistical significance in the enrichment of GO and KEGG pathways.

Quantitative reverse tanscriptase (qRT)–PCR analysis of DEGs was used to validate the RNA-seq data. cDNA synthesis and qRT-PCR were carried out according to the methods used in Zheng et al. (2017). The relative expression level of each gene was calculated using the 2^–ΔΔCt^ method (Livak and Schmittgen, 2001) and was normalized to the control gene *actin*. Each sample had three technical replicates. The primers used for qRT-PCR are listed in Supplementary Table S1.

### Isoform Structure Analysis

Gene structure analysis of polished isoforms was performed using transcriptome analysis pipeline for isoform sequencing (TAPIS v 1.2.1)^1^. The exon–intron structures for each transcript were predicted, and the numbers of introns were statistically analyzed in a transcriptome level. Newly discovered loci and isoforms were identified by comparing the identified loci and isoforms with the reference genome annotation using the same criterion as for loci and isoform identification. Alternative splicing (AS) events were detected by SUPPA v2.3.1 (Alamancos et al., 2015). Alternative polydenylation (APA) events prediction was then performed by TAPIS.

Fusion transcripts need to meet the following criteria (1) the full-length transcript is mapped on two or more loci in the reference genome; (2) each gene locus has to meet a 10% alignment of the corresponding transcript; (3) alignment coverage between the transcript to the reference genome must be more than 99%; and (4) each mapped locus must be more than 100 kb apart on the reference genome.

### Identification of LncRNA and Transcription Factor Prediction

The isoforms with length ≥200 nt were evaluated using software tools PLEK (predictor of long non-coding RNAs and messenger RNAs (mRNAs) based on an improved k-mer scheme, Li et al., 2014) and CNCI (coding–non-coding index, Liang et al., 2018), to distinguish lncRNAs from mRNAs. The remaining transcripts were conducted BLASTX (*e* value cutoff 1e−10) against the protein database in coding potential calculator (CPC) (Kong et al., 2007), and protein-coding transcripts were filtered out. Further, the remaining isoforms were subjected to Pfam-A and Pfam-B database using hmmscan (Finn et al., 2016). In order to ensure the accuracy of the prediction, the non-coding isoforms predicted by all four methods were considered reliable for further analysis.

The transcription factors (TFs) were predicted using iTAK 1.7a based on the known common wheat TFs annotated by IWGSC v2.2 in the plant TF database PlantTFDB v5.0^2^. A total of 3,606 loci classified into 56 TF families were found in the database. The domains of proteins predicted by newly identified isoforms were searched against the 3,606 annotated protein sequences using hmmscan function of the HMMER software, and proteins with exactly the same included domains were regarded as TFs.

## Results

Relative electrolyte leakage was regarded as the direct marker to reflect the membrane damage by freezing stress in previous studies (Campos et al., 2003; Pu et al., 2019). According to the relative electrolyte leakage and the damage level of seedling (Supplementary Figure S1), we selected leaves treated 4 h at −6°C to conduct RNA extraction and transcriptome sequencing.

### Transcriptome Sequencing Using PacBio Iso-Seq

cDNA libraries from control and freezing-treated samples were prepared and subjected to an SMRT sequencing using the PacBio Sequel platform. Adapts and low-quality reads from the original sequencing data were filtered in SMRTlink v7.0; 30,066,483 (30.10 billion bases) and 31,782,257 (33.99 billion bases) subreads were generated from two samples, which formed 549,012 and 737,027 CCSs, respectively (Table 1). Of these CCSs, 456,422 (75.76%) and 575,154 (78.71%) are FLNC reads, containing 3′ primer, 5′ primer, and the poly(A) tail. The FLNC sequences of the same transcript are clustered using a hierarchical n^∗^log(n) algorithm to obtain a consensus sequence. Then, the obtained consensus sequence was subjected to polish using arrow, and 51,570 and 73,695 polished consensus sequences were finally obtained for subsequent analysis. To make further correction of the FLNC reads, all polished consensus isoforms were corrected using the Illumina short reads in Proovread software (Hackl et al., 2014). The sizes of corrected consensus ranged from 82 to 5,034 and 62 to 5,788 nucleotides with an average read length of 1,180 and 1,207 nucleotides, respectively (Table 1).

**TABLE 1:** Summary of reads from PacBio single-molecule long-read sequencing.

	**Subreads**	**CCS**	**FLNC**	**Polished consensus**	**Corrected consensus**
Number	30,066,483	549,012	456,422	51,570	47,637
	31,782,257	737,027	575,154	73,695	68,366
Mean length	1,002	1,175	1,004	1,015	1,180
	1,102	1,303	1,130	1,122	1,207
N50	1,065	1,298	1,138	1,143	1,278
	1,228	1,466	1,322	1,287	1,455

### Genome Mapping of Isoforms

**FIGURE 1:**
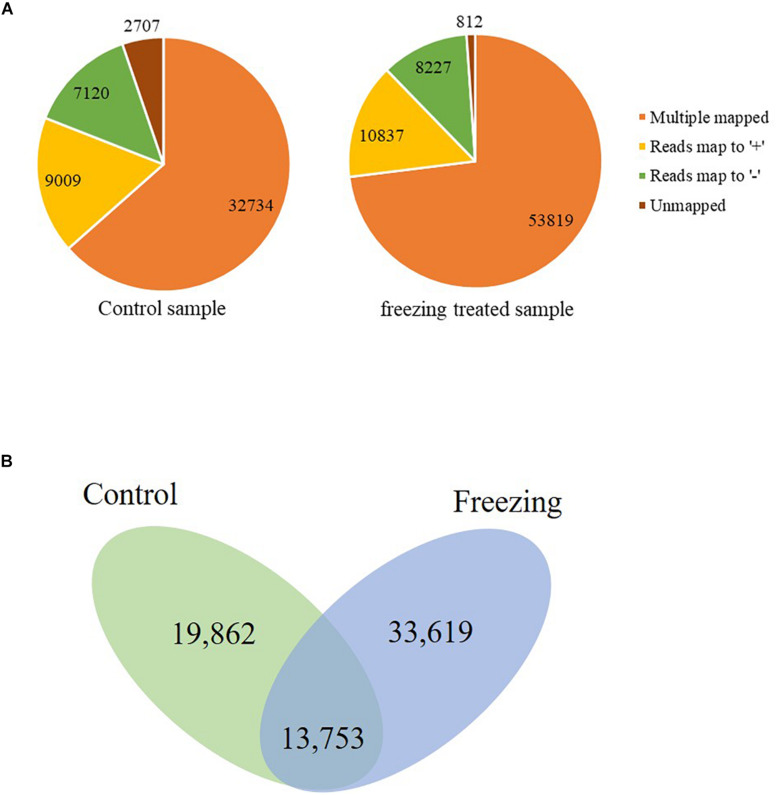
Genome Mapping and Alignment Program mapping reads to the reference sequence. **(A)** Reads classified into four groups (G1–G4). **(B)** Isoforms reserved after clustering and redundancy in TAPIS.

The corrected consensus reads were mapped against the genome sequence of CS using GMAP, and 94.75 and 88.90% reads were mapped to the IWGSC RefSeq v1.1 in the control and freezing-treated sample, respectively. These reads could be divided into four groups (G1–G4, Figure 1A). G1 contained 32,734 reads (63.47%) showing multiple best alignments (with identity and/or coverage values ≥90%) in untreated leaves, while 53, 819 (73.03%) in cold treated sample. G2 included 9,009 (control, 17.47%) and 10,837 (treated, 14.71%) reads exhibiting mapping to “+” strand of the draft genome contigs. G3 consisted of 7,120 reads (control, 13.81% of the total) and 8,227 reads (11.16%) mapping to “−” strand of the draft genome contigs, isoforms of G2 and G3 were mapped to one unique location with higher than 90% coverage and identity. Finally, G4 contained 2,707 reads (5.25%) and 812 (1.1%) with no significant mapping to the draft genome in the untreated and freezing-treated leaves, respectively. Transcripts spanning two or more genes are removed from downstream splice isoform analysis; however, they likely represent misannotations in the gene models. Final high-quality isoforms were obtained by further corrections, clustering, and redundancy of the consensus sequence using TAPIS software (Abdel-Ghany et al., 2016), resulting in 33,615 (control) and 47,372 (freezing-treated) isoforms (Figure 1B).

### Novel Isoforms and Gene Loci Discovering

**FIGURE 2:**
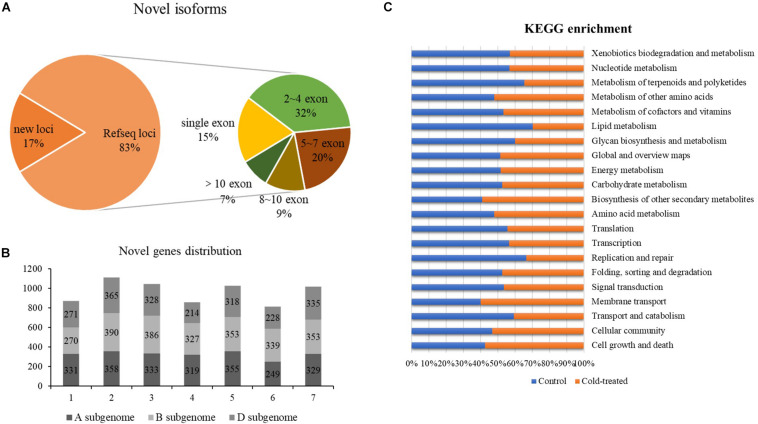
Newly discovered transcripts and genes from PacBio. **(A)** Ratio of novel isoforms mapped to RefSeq and exon number distribution. **(B)** The new genes distribution among A, B, and D subgenomes. **(C)** Functional annotation of new isoforms in Kyoto Encyclopedia of Genes and Genomes (KEGG) database.

Based on the mapping results of the isoforms and the reference genome IWGSC RefSeq v1.1, the reads aligned to the unannotated regions of the reference genome file are defined as new loci and aligned to different exons in the known genes are defined as new isoforms; 4,182 and 4,255 newly discovered genes were identified from control and freezing-treated samples, respectively. In total of the two samples, 57,667 newly discovered isoforms were identified, and 9,700 of them (16.82%) were encoded by 6,889-gene loci, which were not annotated in the IWGSC RefSeq v1.1, and 47,967 novel isoforms (83.18%) were from 20,086 annotated gene loci (Figure 2A). One exon was found in 8,886 (15.41%) transcripts, and the transcript number with ∼2 to 4 exons was dominant, accounting for 31.93% (18, 413), whereas transcripts with more than 10 exons were 3,943 (6.83%) (Supplementary Table S2 and Figure 2A).

We analyzed the chromosomal distribution of the newly identified isoforms encoded by known loci according to the RefSeq annotation, and results showed that more novel isoforms were identified in chromosome group 2 (1,113, 16.16%), group 3 (1,047, 15.20%), group 5 (1,026, 14.90%), and group 7 (1,017, 14.76%). Among the subgenomes, B subgenome shared more newly identified isoforms compared to A and D (Figure 2B).

Further, we compared the newly discovered genes between control and treated samples; 5,341 different genes were identified. Among these genes, 2,634 genes could only be found in the untreated sample, and 2,707 of them specially identified in freezing-treated sample; 1,677 different genes were functional annotated in GO and KEGG (Supplementary Table S3 and Figure 2C).

### Identification of Freezing-Response Genes and Alternative Splicing Events in Wheat Seedling

After 4 h of freezing stress at −6°C, a total of 22,817 DEGs were identified at thresholds of FDR ≤ 0.01 and |log2 fold change|≥1, of which 12,968 showed up-regulation and 9,849 down-regulation.

**FIGURE 3:**
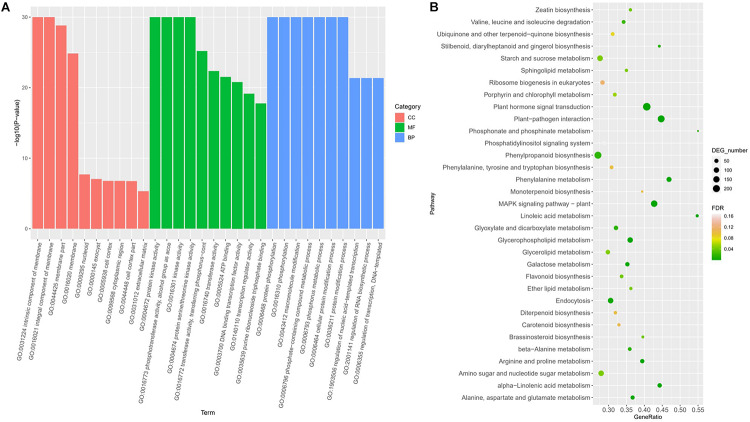
Functional classifications of DEGs under freezing stress using GO analysis **(A)** and KEGG ontology database **(B)**.

Gene Ontology enrichment revealed the terms significantly involved in freezing response (Figure 3A). In the classification of cell components, there are four items (GO: 0031224, GO: 0016021, GO: 0044425, and GO: 0016020) that are significantly enriched, all of which are related to membrane components, indicating that genes involved in the cell membrane system account for a higher proportion in response to freezing damage, and maintaining the integrity of the cell membrane system is very critical in freezing resistant of wheat. Among the molecular functions, the most significant enrichment is the kinase activity (protein kinase activity and protein serine kinase activity) and phosphotransferase activity, indicating that encoding enzymes play a key role in the freezing injury response mechanism of wheat. In biological processes, the most significant enrichment is protein phosphorylation, macromolecule modification, and phosphate complex metabolism. Kyoto Encyclopedia of Genes and Genomes pathway annotation revealed that DEGs were enriched to the plant hormone signal pathway, MAPK signaling, and plant–pathogen interaction pathway (Figure 3B).

ICE-CBF-COR pathway is associated with cold tolerance in crops. Among the 37 *CBF* genes that have been identified in wheat (Guo et al., 2019), 24 of them were up-/down-expressed under freezing stress. Genes that encode low temperature–induced proteins are identified; six genes encoding COR (cold response proteins, TaCOR1b, 1d, 2a, 3b, 3d, and 4a), which could be recognized by *CBF* factors, were up-regulated after freezing shock (Supplementary Table S4). The expression differences of important members such as PYL receptor, PP2C (serine-threonine phosphatase 2C), and SnRK2 (SNF1-related protein kinase 2) kinase in the PYR/PYL/RCARs-PP2C-SnRK2 pathway were also identified in Zhoumai 18. In Supplementary Table S5, 100 of 257 PP2C members were differentially expressed, of which nine were down-regulated, and the rest were up-regulated. Nine of the 27 *TaPYL*s and 4 of the 10 *TaSnRK2* members identified were differentially expressed after 4 h of freezing stress at −6°C. Apart from genes involved in ICE-CBF-COR and PYR/PYL/RCARs-PP2C-SnRK2 pathways, 18 and 14 genes, respectively, encoding dehydrins and WCOR proteins are induced by the freezing stress (Supplementary Table S6). To verify the DEGs analysis, 15 of genes mentioned previously were selected (five in the ICE-CBF-COR pathway, five from cold-induced genes, and five from ABA signal pathway) to conduct qRT-PCR validation. Results showed that the expression patterns of the 15 genes followed the same trends with RNA-seq analyses (Supplementary Figure S2).

**FIGURE 4:**
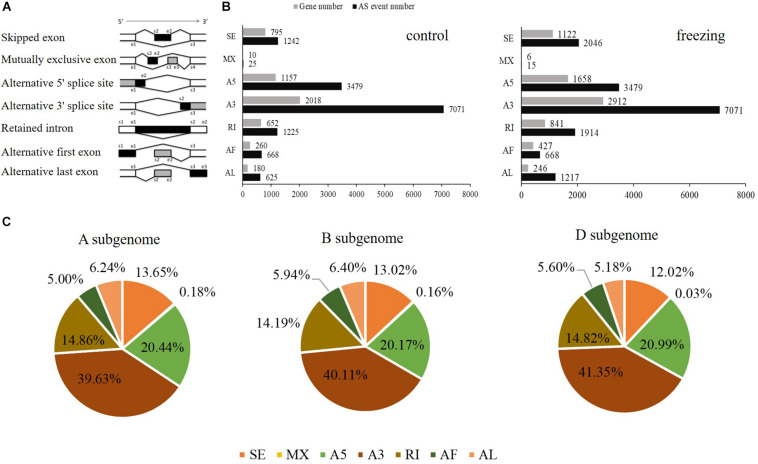
Distributions of different types of alternative splicing events in wheat. **(A)** Schematic representation of seven types of alternative splicing (AS): skipped exon (SE); mutually exclusive exon (MX); alternative 5′ splice site (A5); alternative 3′ splice site (A3); retained intron (RI); alternative first exon (AF); alternative last exon (AL). **(B)** The number of AS events and the number of genes in control and freezing-treated wheat leaves. **(C)** Distributions of different modes of freezing responsive AS events in A, B, and D subgenomes.

SUPPA software was used to identify the AS events. Seven types of AS was identified: skipped exon (SE), Mutually exclusive exon (MX), alternative 5′ splice site (A5), alternative 3′ splice site (A3), retained intron (RI), alternative first exon (AF), and alternative last exon (AL) (Figure 4A). In total, 14,335 AS events within 5,072 genes and 16,410 events within 7,212 genes were identified from the control and stress treatment samples, respectively, accounting for 24.90 and 31.30% of total expressed genes (Figure 4B). There were 3,330/6,445 isoforms located in the 5,943/8,851 known gene loci mainly due to AS. Compared with reference genome, 94 and 144 novel isoforms were located in 43 and 49 novel gene loci based on Iso-seq data of two samples, respectively. Among the seven main types of AS, A3 was the most abundant (39.63%–41.35%) AS events, followed by A5 (20.17%–20.99%), RI (14.19%–14.86%), and SE (12.02%–13.65%) (Figure 4B). The AS events distributed among the A, B, and D subgenomes are comparable, which was consistent with previous reports (Figure 4C; Liu et al., 2017).

### TF Identification

**FIGURE 5:**
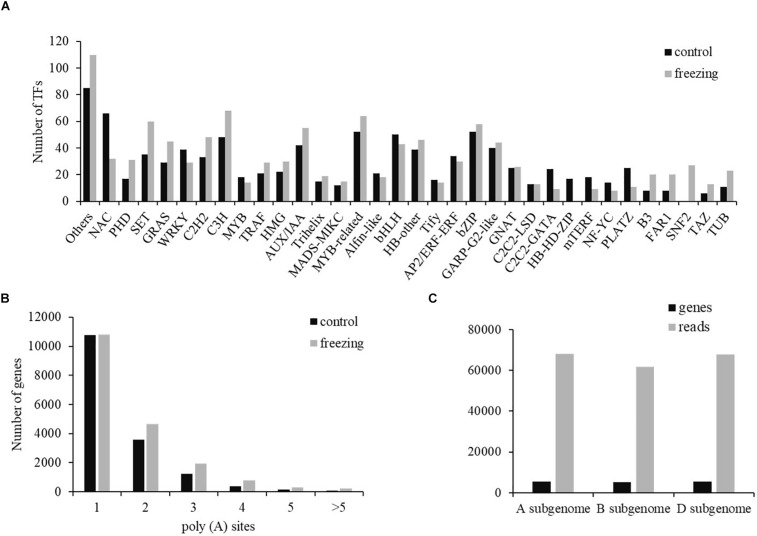
Transcription factor and poly(A) prediction. **(A)** Number of TFs from 34 families predicted by SMRT in both samples. **(B)** Gene distribution of different number of poly(A) sites. **(C)** Distribution of genes and transcripts with poly(A) sites among A, B, and D subgenomes.

Two types of regulatory proteins, TFs and transcriptional regulators (TRs), were identified using the iTAK software. We predicted 955 (684 TF and 271 TR) and 1,089 (699 TF and 390 TR) isoforms from 52 TF families and 21 TR families in control and freezing samples, respectively (Supplementary Table S3). Compared with TFs in the database, 740 transcripts (77.49%) in control sample and 1,036 transcripts (95.13%) in freezing treatment were demonstrated to be new transcripts. Figure 5 shows the number of isoforms from top 34 families predicted in the control and cold-treated leaves, respectively. When suffering from freezing, more TF isoforms belonging to PHD, SET, GRAS, C2H2, and C3H families were detected (Figure 5A). Among these DEGs identified under freezing stress, TFs involved in cold response were annotated, including 128 MYB/MYB-related, 115 NAC, 96 WRKY, 50 AP2/ERF/CBF, and 14 bZIP members of TFs.

### APA Identification

Most genes of eukaryotes can produce a variety of different mRNA 3′ ends through variable APA (Abdel-Ghany et al., 2016). Studies have shown that poly(A) sites are dynamically regulated during tissue development and are automatically regulated by environmental stimuli. Using the TAPIS pipeline to analyze the heterogeneity in 3′ end formation of all transcripts, poly(A) sites in wheat genome were detected. Of the 16,184 genes identified by sequencing, 202,973 reads with 24,427 poly(A) sites were detected. Among these genes, 10,778 genes (66.60%) have single poly(A) sites (Figure 5B). The average number of transcripts with poly(A) sites aligned to one certain annotated gene is 12.54. Transcripts with poly(A) site distributed comparably in A and D subgenomes, with the number in B subgenome ∼6,000 less than the other two subgenomes (Figure 5C).

### LncRNA and Fusion Transcript Identification

**FIGURE 6:**
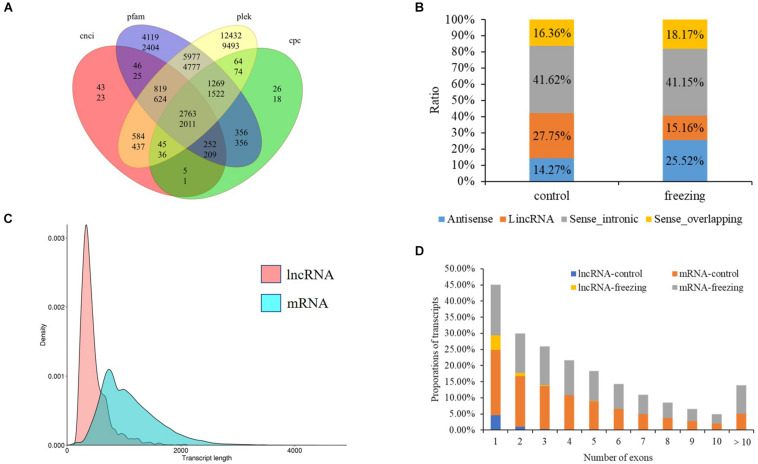
LncRNA predicted in this study. **(A)** Venn diagram of lncRNA number predicted by four methods (upper number shows lncRNA predicted in control sample, and the number below shows lncRNA predicted in freezing-treated sample). **(B)** Classification of lncRNAs. **(C)** Density and transcript length distributions of lncRNAs and mRNAs. **(D)** Comparison of exon numbers between lncRNA and mRNA in freezing-treated and untreated samples.

**FIGURE 7:**
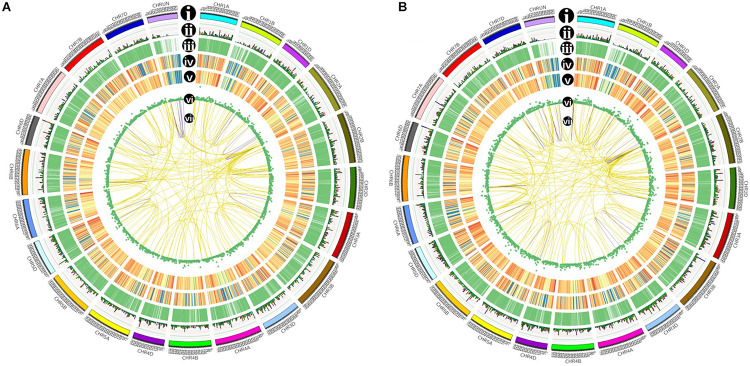
Isoforms distribution of control **(A)** and cold-treated **(B)** wheat leaves in CIRCOS visualization. (i) Karyotype of wheat chromosomes; (ii) alternative splicing (AS) sites in wheat genome; (iii) comparison of APA sites in wheat genome; (iv) density of novel transcript from PacBio data. The closer to red, the higher density the color represents and the closer to blue, the lower density the color represents; (v) density of newly discovered gene from PacBio data; (vi) LncRNA distribution; (vii) linkage of fusion transcripts. Purple color indicates intrachromosomal fusion; yellow indicates interchromosomal.

After filtering through the four methods PLEK, CNCI, CPC, and pfam, 2,763 (control sample) and 2,011 (freezing treated) lncRNAs were identified (Figure 6A). According to the position they aligned against the reference sequence (Sun et al., 2013), these predicted lncRNAs were classified into four groups: 14.27 and 25.51% antisense, 27.75 and 41.15% lincRNA, 41.62 and 15.16% sense_intronic, and 16.36 and 18.17% sense_overlapping (Figure 6B). The ratio of number between sense_intronic and sense_overlapping was comparable in the treated and untreated leaves; the antisense lncRNA accounted for a higher ratio in freezing-treated leaves (Figures 6C,D). More interchromosome fusion events were found than intrachromosome (Figures 7A,B); the results are consistent with the fusion events in maize B73 and red clover (Wang et al., 2016; Chao et al., 2018).

## Discussion

### Utility of PacBio Transcriptome Sequencing for Obtaining New Information in Common Wheat Genome

The full-length transcriptome is based on the PacBio Sequel three-generation sequencing platform, which can directly obtain complete transcripts containing 5′, 3′UTR, and poly(A) tails without assembling short transcriptomic reads (Liu Q. et al., 2019). The long read sequencing provides significant benefits to identify and differentiate homologous genes among subgenomes from polyploidy species, such as the hexaploid wheat (Dong et al., 2015; Liu Z. et al., 2019). Utility of PacBio transcriptome sequencing was not only for discovering new genes, transcripts, and AS events to improve genome annotation, but also for increasing the accuracy of RNA-seq quantification with isoform-level resolution, thereby accurately analyzing structural information such as lncRNA and fusion genes of reference genomic species (Xiao et al., 2017). In this work, we performed a full-length transcriptome analysis of wheat leaves under freezing stress, providing valuable insight for future to reveal the regulatory network of cold adaptations. Despite its strong advantages, the technology has its own limitations: high error rates and relatively low throughput, making it difficult to provide quantitative information about gene expression levels at this point. Illumina short reads sequencing were conducted to verify and quantify genes/isoforms identified in PacBio sequencing.

Compared with transcripts from the IWGSC RefSeq v1.1, 57,667 novel isoforms were discovered by PacBio SMRT sequencing, which were annotated 21,672 known gene loci, as well as 3,399 novel gene loci. Consistent with the transcriptome sequencing in Xiaoyan 81 using PacBio RSII platform (Dong et al., 2015), in this work 3,026 more loci were newly annotated in subgenome B than subgenomes A and D (Figure 2B). This may be caused by the larger size of chromosomes from subgenome B than those from subgenomes A and D in common wheat. These newly discovered isoforms and genes provide valuable information for the common wheat genome annotation and transcriptome research, as well as gene clone and function analysis. Previous studies have indicated that extensive AS events are involved in development and in response to biotic and abiotic stresses in plants (Mei et al., 2017). Liu et al. (2017) discovered AS events in wheat seedling during drought, heat, and their combination stress, proposing that transcriptional regulation plays a major role in drought stress, and AS coordinated with transcriptional regulation contributes to heat and their combination stress. Using the PacBio sequencing, our analysis detected 138,140 junction sites in 51,570 isoforms, approximately 16% of which were newly discovered transcripts in untreated common wheat leaves. After treatment in −6°C, we detected nearly 10,000 more transcripts than the control group sample, and some of these transcripts may be caused by AS events. Considering that we used only one tissue under one timepoint treatment, the number of AS events may be underestimated. Cold response at multiple timepoints in different tissues (root, young spike, and crown) will be analyzed to reveal more AS transcripts-harboring genes.

### Key Signaling Pathways Participated in Freezing Resistance in Wheat Seedling

In plants, ABA signals are well known to take part in regulation of abiotic tolerance, especially drought. The PYL receptor, PP2C, and SnRK2 are important elements of ABA signal transduction pathway. As an important class of protein phosphatases, PP2C regulates plant stress signals by catalyzing the dephosphorylation and phosphorylation reactions of proteins downstream. SnRK2 protein kinases can bind to bZIP-like TFs and play a key role in plant stress signal transduction pathways. In our study, we identified 9 *PYL*, 100 *PP2C*, and 4 *SnRK2* genes showing differential expression to freezing stress in Zhoumai 18. Under freezing shock, the *PP2C* and *SnRK2* genes were activated and regulate expressions of cold-inducible genes, which is consistent with the significant enrichment of phosphatases and protein kinases activity in GO analysis. Previous studies showed that *TaPP2C* members in clade A can respond to drought, salt, ABA, and other stresses and could interact with *TaSnRK2*s (Liu et al., 2013; Yu et al., 2019). It has been proved that related elements on the ABA signaling pathway play an important role in resisting cold stress in wheat. *TaSnRK2.7* gene is specifically expressed in roots and participates in many physiological and biochemical processes, such as regulating carbohydrate metabolism, reducing osmotic potential, and promoting root development. Within 24 h of low-temperature treatment, the expression of *TaSnRK2.7* gene reached the highest level. The overexpression of *TaSnRK2.7* significantly increased the resistance of *Arabidopsis* to drought, salt, and low-temperature stress (Zhang et al., 2010).

### The TFs and Cold Responsive Genes Identified Are Colocated Within Freezing Tolerance-Related Quantitative Trait Loci (QTL)

Although tolerance to subzero temperatures is often critical to winter survival, the genetic dissection of freezing tolerance of wheat has been rather limited (Kruse et al., 2017). The *Vrn* loci located on chromosome 5 are known to have pleiotropic effect to affect both the vernalization response and frost hardiness of winter wheat (Koemel et al., 2004). Maps close to *Vrn-A1*, frost-resistance loci, *Fr-A1*, and *Fr-A2* have also been reported associated with freezing tolerance (Vágújfalvi et al., 2003). The *Fr-A2* locus approximately maps 30 to 46 cM proximal to *Vrn-1A* and has been shown to carry a cluster of several *CBF* (*C*-repeat binding factor) genes that upregulate many cold-responsive genes under low temperatures (Lee et al., 2015; Würschum et al., 2017). Apart from these major loci on 5A, a series of minor QTLs have been reported to affect winter hardiness and freezing resistance (Båga et al., 2007; Case et al., 2014). Differentially expressed genes identified in this study were mapped to wheat chromosome and collocated with the known QTLs; a total of 10 DEGs under freezing stress were found colocated within the frost tolerance related QTL *Fr-A2*. Under freezing stress, expressions of *TraesCS5A02G291200* (NAC), *TraesCS5A02G296800* (C2H2), *TaCBF8a*, and *TaCBF14a* were decreased, and *TaCBF6a*, *TaCBF9a*, *TaCBF10a*, *TaCBF13a*, and *TaCBF15a* were increased (Supplementary Table S7), indicating these TFs have a potential role in frozen tolerance. The candidate genes associated with tolerance could be utilized for wheat engineering in resistance breeding.

## Data Availability Statement

The datasets generated for this study can be found in the raw data about Illumina and Pacbio sequence has been uploaded to NCBI with a SRA number PRJNA592401.

## Author Contributions

XZ, JuZ, TX, and JiZ designed the work. XZ, MS, JW, and NY performed the experiments in laboratory. KW conducted the field work. JX performed the Illumina short reads sequencing and qRT-PCR validation. JX and CW analyzed the data. XZ drafted the manuscript. All authors read and approved the final manuscript.

## Conflict of Interest

The authors declare that the research was conducted in the absence of any commercial or financial relationships that could be construed as a potential conflict of interest.

## Footnotes

1 https://bitbucket.org/comp_bio/tapis

2 http://planttfdb.cbi.pku.edu.cn/index.php
