# Supplementary figures and images for: Isoform Sequencing Provides Insight Into Freezing Response of Common Wheat (Triticum aestivum L.)
Source: Front Genet. 2020 Jun 11;11:462. doi: 10.3389/fgene.2020.00462 (PMC7300213; doi:10.3389/fgene.2020.00462)

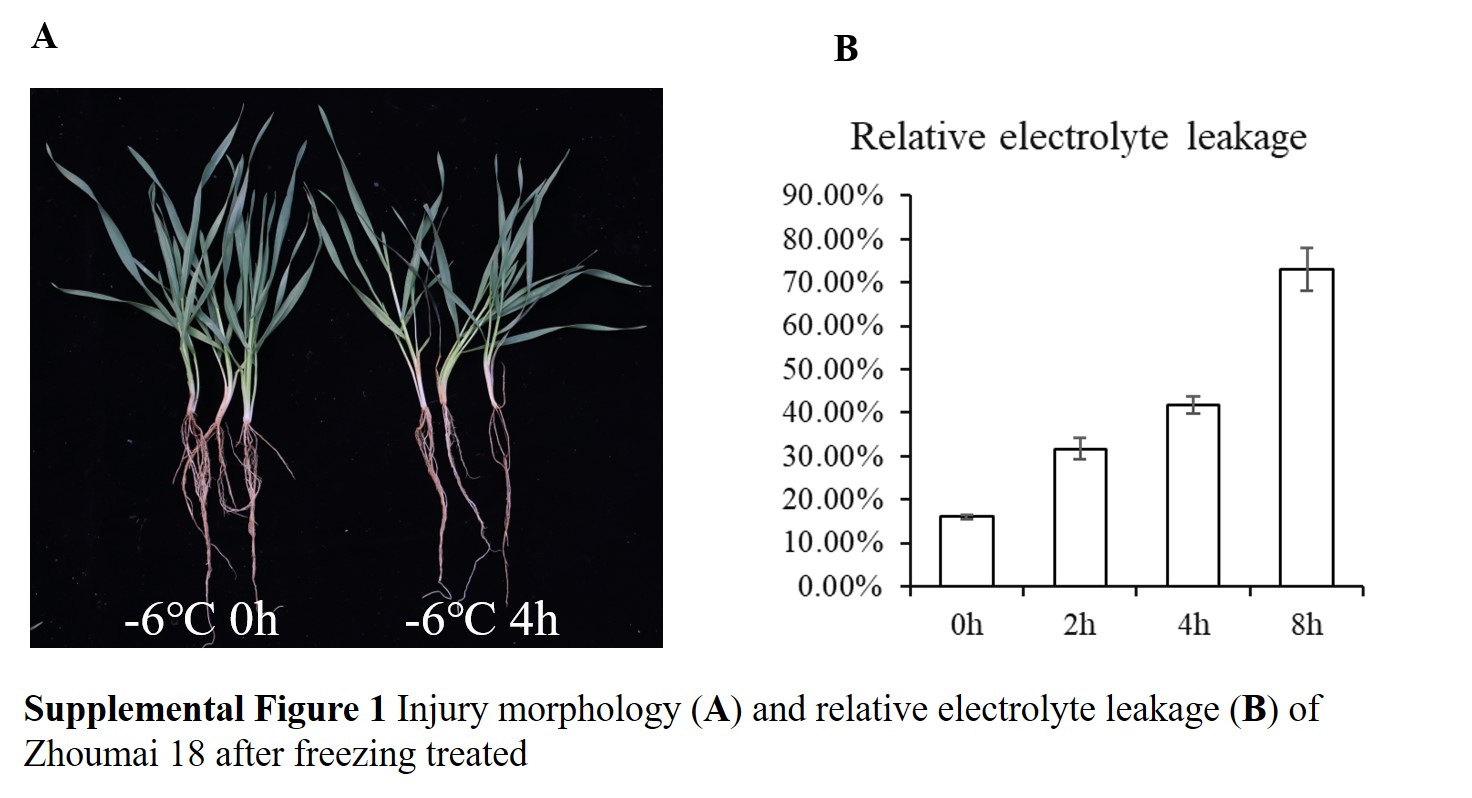

Supplement: Supplementary file 1 [file Image_1.jpg]

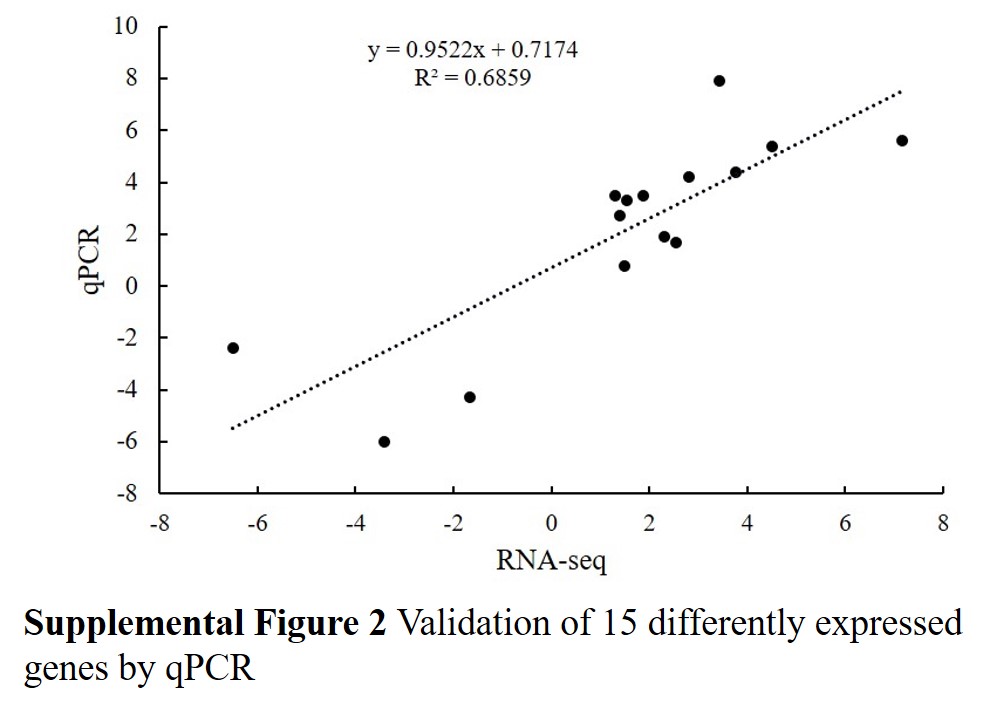

Supplement: Supplementary file 2 [file Image_2.jpg]
